# Deep learning models for MRI-based clinical decision support in cervical spine degenerative diseases

**DOI:** 10.3389/fnins.2024.1501972

**Published:** 2024-12-06

**Authors:** Kai-Yu Li, Zhe-Yang Lu, Yu-Han Tian, Xiao-Peng Liu, Ye-Kai Zhang, Jia-Wei Qiu, Hua-Lin Li, Yu-Long Zhang, Jia-Wei Huang, Hao-Bo Ye, Nai Feng Tian

**Affiliations:** ^1^Department of Orthopedic Surgery, Second Affiliated Hospital and Yuying Children’s Hospital of Wenzhou Medical University, Wenzhou, China; ^2^Renji College of Wenzhou Medical University, Wenzhou, China

**Keywords:** deep learning, convolutional neural network, magnetic resonance imaging, cervical spine degenerative diseases, clinical decision

## Abstract

**Purpose:**

The purpose of our study is to develop a deep learning (DL) model based on MRI and analyze its consistency with the treatment recommendations for degenerative cervical spine disorders provided by the spine surgeons at our hospital.

**Methods:**

In this study, MRI of patients who were hospitalized for cervical spine degenerative disorders at our hospital from July 2023 to July 2024 were primarily collected. The dataset was divided into a training set, a validation set, and an external validation set. Four versions of the DL model were constructed. The external validation set was used to assess the consistency between the DL model and spine surgeons’ recommendations about indication of cervical spine surgery regarding the dataset.

**Results:**

This study collected a total of 756 MR images from 189 patients. The external validation set included 30 patients and a total of 120 MR images, consisting of 43 images for grade 0, 20 images for grade 1, and 57 images for grade 2. The region of interest (ROI) detection model completed the ROI detection task perfectly. For the binary classification (grades 0 and 1, 2), DL version 1 showed the best consistency with the spine surgeons, achieving a Cohen’s Kappa value of 0.874. DL version 4 also achieved nearly perfect consistency, with a Cohen’s Kappa value of 0.811. For the three-class classification, DL version 1 demonstrated the best consistency with the spine surgeons, achieving a Cohen’s Kappa value of 0.743, while DL version 2 and DL version 4 also showed substantial consistency, with Cohen’s Kappa values of 0.615 and 0.664, respectively.

**Conclusion:**

We initially developed deep learning algorithms that can provide clinical recommendations based on cervical spine MRI. The algorithm shows substantial consistency with experienced spine surgeons.

## 1 Introduction

As aging progresses, degenerative cervical spine diseases are affecting an increasing number of people (Todd, 2011; Oglesby et al., 2013). As people age, it is common to present with radiographic evidence of cervical spine degeneration, of which not all will show significant clinical signs. Most symptomatic patients with cervical spondylosis can find relief through lifestyle changes or non-surgical treatments, such as physical therapy, cervical traction, and oral analgesics (Lannon and Kachur, 2021). However, patients who experience severe neurological symptoms and show significant spinal cord or nerve root compression on imaging often require surgical intervention (Soufi et al., 2022; Wilson et al., 2017; Badhiwala et al., 2020).

For degenerative cervical spine diseases, MRI is the preferred imaging modality because it can display the neural tissue, bone, and ligament structures with high resolution (Khan et al., 2023). In T2-weighted MRI, the imaging findings of cervical spondylosis include nerve root compression, osteophyte formation, spinal cord compression, disk herniation, and vertebral slippage (Nouri et al., 2016). While MRI serves as an important basis for selecting treatment options, diagnosing cervical spondylosis is relatively straightforward. However, for non-specialist or inexperienced clinicians, assessing the severity of nerve root or spinal cord compression and determining whether surgical intervention is necessary is a challenge. Most radiologists can provide diagnostic reports based on MR images, but patients cannot easily ascertain from the report alone whether surgery is required or if they need to seek care at a higher-level hospital.

In recent years, deep learning has gradually been popularized in the field of spine surgery, especially in diagnostic imaging (Ong et al., 2022; Haim et al., 2024; Qu et al., 2022). In previous studies, deep learning models, due to their excellent image analysis capabilities, have helped improve the diagnostic efficiency and accuracy of clinicians (Qu et al., 2022; Yang et al., 2024). There have been no previous studies using DL models for clinical decision making in cervical spine disease. The aim of our study is to develop MRI-based deep learning models and analyze their degree of consistency with treatment recommendations provided by spine surgeons in our hospital regarding degenerative cervical spine disorders.

## 2 Materials and methods

This retrospective diagnostic study obtained approval from the Institutional Review Board of the Second Affiliated Hospital of Wenzhou Medical University and did not require written informed consent.

### 2.1 Case selection and data set collection

Data collection consisted of MRI T2-weighted cross-sectional images of the intervertebral disks at the cervical levels C3-C7, with four images per patient. The MRI images were obtained using an Avanto (Siemens Healthineers, Forchheim; 1.5 T) machine, equipped with an eight-channel receiving coil. The dataset primarily included patients who visited our hospital between July 2023 and July 2024 due to clinical symptoms related to cervical spondylosis (such as radiating pain in the upper limbs, loss of hand dexterity, gait and balance disturbances, etc.) (Nouri et al., 2017; Kim et al., 2013). Table 1 presents the inclusion and exclusion criteria. The dataset was divided into training, validation, and external validation sets, with the training and validation sets randomly allocated.

**Table 1:** Summary of study inclusion and exclusion criteria.

Criteria for inclusion and exclusion
Inclusion Patients hospitalized due to degenerative cervical spine disorder. Symptoms associated with cervical degenerative disease (neck pain, upper limb pain, gait instability, and muscle weakness). MRI diagnosis of cervical degenerative disease at our hospital. Exclusion Poor image quality. Spinal fracture, infection, deformity, tumor, or inflammatory spondyloarthropathy. Previous cervical spine surgery.

There were three categories of treatment recommendations (recommendations for patients, divided from non-invasive to invasive): low surgical recommendation level (grade 0), where surgery was not recommended for the time being; medium surgical recommendation level (grade 1), where conservative treatment was recommended, and surgery can be considered if conservative treatment fails or if the patient has a strong desire for surgery; and high surgical recommendation level (grade 2), where there was a high risk of neurological deficits, and immediate surgical treatment was recommended. The surgical plans for all selected patients were determined through departmental discussions (including at least one chief spine surgeon and two attending spine surgeons), and corresponding treatment levels were assigned to each patient.

### 2.2 DL model establishing

The DL model was divided into two parts, which were the region of interest (ROI) auto-detection model and the convolutional neural network (CNN) classification model. The ROI auto-detection model was mainly used to extract the ROI region (including the cervical spinal canal). The ROI auto-detection model consisted of the Faster R-CNN and MobileNet as the framework, and the data labels were the coordinates of the upper-left and lower-right corners of the ROI region. Two spine surgeons (one with 5 years of clinical experience and the other with 10 years of clinical experience) completed the ROI label formulation task.

The classification model used four types of CNN models as a framework (MobileNet, EfficientNet, Mnasnet and Regvgg) and used a validation set for initial validation of the model. The selection of models was based on the following criteria: (1) The model was sourced from the timm library. (2) A lightweight CNN model suitable for small grayscale images was chosen. (3) The model had to achieve a consistency rate of over 70% in the internal validation set.

**Figure 1:**
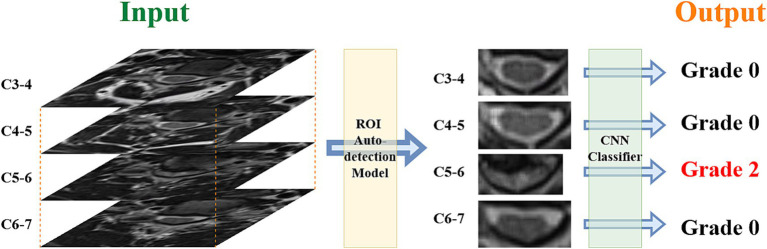
Process from input data to output categories.

Deep learning models were constructed using the PyTorch framework, using a pre-trained timm model as the backbone network, combined with data augmentation techniques to process the training data. Data augmentation techniques mainly included randomly flipping images horizontally, randomly adjusting the brightness and contrast of images, which effectively expanded the available training dataset and enhanced the robustness of the model. The models used the cross-entropy loss function and AdamW optimizer for parameter optimization, while the learning rate scheduler was used to dynamically adjust the learning rate. Mixed-precision training was introduced during the training process to improve the training speed and numerical stability. During the training process, metrics such as loss and accuracy on the training and validation sets were recorded, and the best model was saved. The whole process included steps such as data loading, model construction, training cycle, validation, saving the best model, and selecting the best DL model for the classification task. Figure 1 shows the process from MR images to model output results.

### 2.3 DL model performance validation

The external validation set consisted of newly admitted patients from May 2024 to July 2024 (meeting inclusion and exclusion criteria). The evaluation of the ROI selection model was conducted through visual analysis, carried out by the two spine surgeons who formulated the ROI labels. The validation set was used for the preliminary evaluation of the trained model. A consistency rate greater than 75% was considered as the completion of training. The trained ensemble CNN model was then evaluated on the external validation set. Finally, the evaluation results were compared with the assessments made by spine surgeons for consistency testing.

### 2.4 Statistical analysis

The DL model was implemented using PyTorch version 2.1.0. Both used open-source code, available on GitHub (San Francisco, CA). All analyses were conducted using SPSS (version 25.0; IBM, Armonk, NY, United States), with differences considered statistically significant (*p* < 0.05). The consistency test comparing the model with specialist physicians was performed using Cohen’s Kappa. The levels of consistency for Cohen’s Kappa were defined as follows: less than 0.2 indicates poor consistency; 0.21–0.4 indicates fair consistency; 0.41–0.6 indicates moderate consistency; 0.61–0.8 indicates substantial consistency; and greater than 0.8 indicates almost perfect consistency. All code has been uploaded to https://github.com/leekaiyu123/MRI-CS.

## 3 Results

### 3.1 Patient data

A total of 756 MR images were collected for this study, sourced from 189 patients. Among these, the training set consisted of 490 images, and the validation set comprised 146 images. There were 279 images for grade 0, 159 images for grade 1, and 198 images for grade 2 in the training and validation set.

### 3.2 ROI detection model

The ROI detection model was trained using 110 MR images. Visual analysis was conducted in the external control set. All ROI regions in the external validation set were perfectly captured.

### 3.3 CNN classification model

A total of four CNN classification models were trained, namely DL model 1, DL model 2, DL model 3, and DL model 4. DL model 1 was primarily built using mnasnet_small and achieved 77.4% consistency after seven training epochs. DL model 2 was primarily built using mobilenetv3_small_050 and achieved 76.7% consistency after 34 training epochs. DL model 3 was primarily built using efficientnet_b0 and achieved 76.1% consistency after 10 training epochs. DL model 4 was primarily built using resnest14d and achieved 76.0% consistency after 28 training epochs.

### 3.4 Combined CNN model validation

The external validation set included 30 patients, with a total of 120 MR images, where there were 43 images for grade 0, 20 images for grade 1, and 57 images for grade 2. The average age of the patients was 56 years ±15 years (34–86), with 19 males and 13 females.

The results analysis was divided into binary classification and three-class classification. The binary classification included cases requiring surgery (grade 1, 2) and cases not requiring surgery (grade 0). In the binary classification, DL version 1 showed the best consistency with the spine surgeons, achieving a Cohen’s Kappa value of 0.874 (CI: 0.661, 1.000). DL version 4 also achieved nearly perfect consistency, with a Cohen’s Kappa value of 0.811 (CI: 0.580, 1.000). DL version 2 and DL version 3 demonstrated substantial consistency, with Cohen’s Kappa values of 0.761 (CI: 0.527, 0.991) and 0.746 (CI: 0.516, 0.977), respectively.

In the three-class classification, DL version 1 showed the best consistency with the spine surgeons, achieving a Cohen’s Kappa value of 0.743 (CI: 0.575, 0.910). DL version 2 and DL version 4 also demonstrated substantial consistency, with Cohen’s Kappa values of 0.615 (CI: 0.431, 0.799) and 0.664 (CI: 0.489, 0.839), respectively.

**Figure 2:**
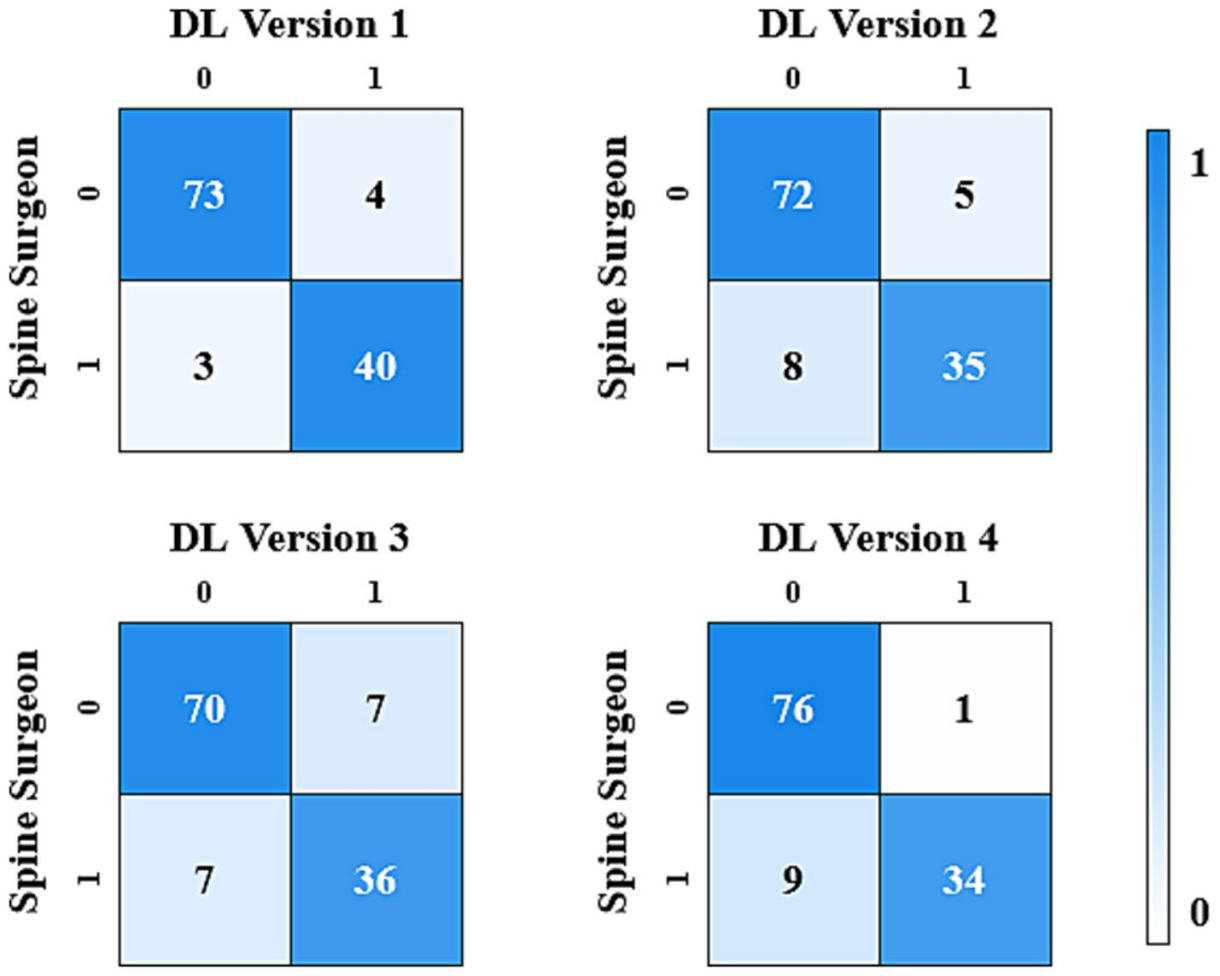
Confusion matrix for the binary classification made by spinal surgeons and DL models.

**Figure 3:**
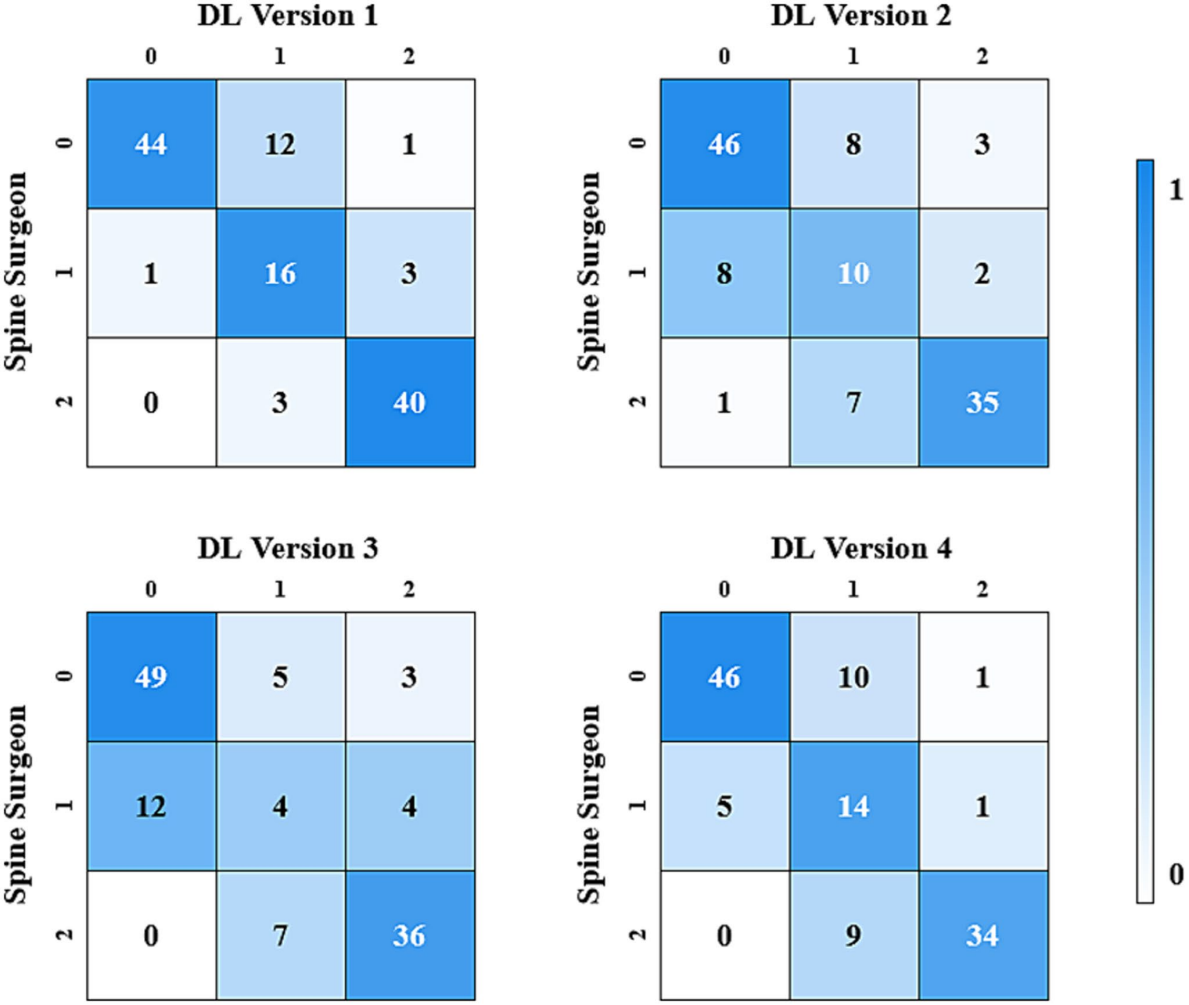
Confusion matrix for the three-class classification made by spinal surgeons and DL models.

The results of the consistency test between the four versions of the ensemble model and the assessments made by spine surgeons are listed in Table 2. Figures 2, 3 gave the confusion matrices for binary and three-class classification of the ensemble model compared with the assessment of spine surgeons, respectively.

**Table 2:** Kappa scores and confidence intervals for the Four DL models.

		Dichotomous gradings	Three gradings		
	Architecture	Cohen’s Kappa	*p* value	Cohen’s Kappa	*p* value
DL model version 1	Mnsnet	0.874 (0.661, 1.000)	<0.001	0.743 (0.575, 0.910)	<0.001
DL model version 2	Mobilenet	0.761 (0.527, 0.991)	<0.001	0.615 (0.431, 0.799)	<0.001
DL model version 3	Efficientnet	0.746 (0.516, 0.977)	<0.001	0.575 (0.382, 0.768)	<0.001
DL model version 4	Regvgg	0.811 (0.580, 1.000)	<0.001	0.664 (0.489, 0.839)	<0.001

Data in parentheses are 95% CIs.

## 4 Discussion

In this study, we preliminarily built DL models for clinical decision making in cervical degenerative diseases. The model demonstrated a high degree of consistency in clinical decision making with experienced spine surgeon.

There are previous studies based on MRI to diagnose cervical spine degenerative diseases. Yi et al. (2023) proposed a DL model based on T2-weighted MR images for detecting lumbar and cervical spine degenerative diseases. The model was evaluated on an independent cervical spine MRI dataset and achieved F1 scores of 0.931 and 0.919 on sagittal and axial MR images, respectively, showing good generalization ability. The model can be used to aid in diagnosis, but cannot give specific treatment recommendations.

Previous studies also explored DL models to guide the decision of whether surgery was needed. Suzuki et al. (2024) developed a deep learning algorithm based on a CNN model to automatically detect lumbar spinal stenosis requiring surgical treatment in lumbar X-ray images. This model performed excellently in detecting surgical cases of lumbar spinal stenosis, achieving an internal validation AUC of 0.85–0.89 and a detection accuracy of 79–83%. The external validation AUC was 0.90, with an accuracy of 82%. X-rays, as two-dimensional images, have many limitations and cannot accurately assess the degree of nerve compression. MRI is very important imaging data for evaluating whether surgery is necessary.

According to the AOSpine North America and CSRS guidelines, as well as recommendations from the WFNS Spine Committee, surgical treatment was recommended for moderate to severe degenerative cervical myelopathy (mJOA score < 15). No clear guidelines were established for mild of degenerative cervical myelopathy (mJOA score ≥ 15) (Parthiban et al., 2019). In clinical practice, the decision to perform surgery was typically made by spine surgeons based on objective evidence and subjective judgment, which included the patient’s imaging, clinical signs, history, and physical examination. Of course, MRI also served as an important indicator for assessing whether a patient required surgery (Nouri et al., 2017; Severino et al., 2020).

In this study, combined CNN models were used to classify MR images. We used Faster R-CNN as the ROI detection model, Faster R-CNN has the advantages of being able to efficiently generate candidate frames for the target region, and also has a strong generalization ability to maintain efficient detection performance in a variety of real-world applications and can be used in conjunction with various types of convolutional neural networks. Faster R-CNN well accomplished the ROI detection task in this study. Among the CNN classification models, the DL version 1 built using Mnasnet as the framework demonstrated the highest consistency with spine surgeons, showing almost perfect agreement when evaluating the categories of treatment recommendations (Cohen’s Kappa >0.8). As a preliminary exploration of using DL models to guide clinical strategies, this study may provide insights for future DL models to transition from clinical assistance to clinical guidance. At this stage, the proposed DL model can serve as a tool for healthcare professionals who are not spine surgeons to provide recommendations on whether a referral to a spine surgeon is indicated. However, the model still needs to be further verified. If multi-center and large-scale studies can be continued. The model may have the potential to alert patients to prevent serious neurological complications and provide surgical plans for specialist physicians.

There were areas for improvement in the DL model developed for this study. First, as mentioned above, the labels for the data did not have specific standards and relied on subjective judgments from spine surgeons. Although all cases in this study were discussed within the department, they were still influenced by the personal habits of the specialists and the varying standards across different hospitals. Second, the model provided diagnostic and therapeutic recommendations based only on the MRI T2 sequence data and did not take into account the rest of the imaging data, the patient’s clinical symptoms, history, and physical examination. Third, in order to promote the model, more institutions and a larger volume of data were needed to accommodate the different equipment used by various organizations. If a standardized labeling system can be established to reduce subjectivity in data labeling and differences between hospitals, while incorporating various imaging data and clinical information, it would be possible to develop personalized treatment plans based on the specific circumstances of the patients.

## 5 Conclusion

We initially developed deep learning algorithms that can provide clinical recommendations based on cervical spine MRI. The algorithm shows substantial consistency with experienced spine surgeons.

## Data Availability

The raw data supporting the conclusions of this article will be made available by the authors, without undue reservation.
